# Could health information systems enhance the quality of Aboriginal health promotion? A retrospective audit of Aboriginal health programs in the Northern Territory of Australia

**DOI:** 10.1186/s12911-020-01300-0

**Published:** 2020-11-03

**Authors:** Nikki Percival, Priscilla Boucher, Kathleen Conte, Kate Robertson, Julie Cook

**Affiliations:** 1 Faculty of Health, Australian Centre for Public and Population Health Research, University of Technology Sydney, UTS Building 10, Level 8, 235-253 Jones Street, Ultimo, Sydney, NSW 2007 Australia; 2 Department of Health, Strategic, Policy and Planning, Northern Territory Government, Darwin, NT Australia; 3 Faculty of Medicine and Health, School of Public Health, Menzies Centre for Health Policy and University Centre for Rural Health, University of Sydney, Sydney, NSW Australia; 4 Department of Health, Top End Health Services, Primary Health Care Outreach Team, Northern Territory Government, Katherine, NT Australia

**Keywords:** Health promotion, Indigenous, Information systems, Health information technology, Quality improvement, Audit, Performance indicators, Aboriginal health, Delivery of healthcare, Northern territory

## Abstract

**Background:**

In Australia, health services are seeking innovative ways to utilize data stored in health information systems to report on, and improve, health care quality and health system performance for Aboriginal Australians. However, there is little research about the use of health information systems in the context of Aboriginal health promotion. In 2008, the Northern Territory’s publicly funded healthcare system introduced the quality improvement program planning system (QIPPS) as the centralized online system for recording information about health promotion programs. The purpose of this study was to explore the potential for utilizing data stored in QIPPS to report on quality of Aboriginal health promotion, using chronic disease prevention programs as exemplars. We identify the potential benefits and limitations of health information systems for enhancing Aboriginal health promotion.

**Methods:**

A retrospective audit was undertaken on a sample of health promotion projects delivered between 2013 and 2016. A validated, paper-based audit tool was used to extract information stored in the QIPPS online system and report on Aboriginal health promotion quality. Simple frequency counts were calculated for dichotomous and categorical items. Text was extracted and thematically analyzed to describe community participation processes and strategies used in Aboriginal health promotion.

**Results:**

39 Aboriginal health promotion projects were included in the analysis. 34/39 projects recorded information pertaining to the health promotion planning phases, such as statements of project goals, ‘needs assessment’ findings, and processes for consulting Aboriginal people in the community. Evaluation findings were reported in approximately one third of projects and mostly limited to a recording of numbers of participants. For almost half of the projects analyzed, community participation strategies were not recorded.

**Conclusion:**

This is the first Australian study to shed light on the feasibility of utilizing data stored in a purposefully designed health promotion information system. Data availability and quality were limiting factors for reporting on Aboriginal health promotion quality. Based on our learnings of QIPPS, strategies to improve the quality and accuracy of data entry together with the use of quality improvement approaches are needed to reap the potential benefits of future health promotion information systems.

## Background

It is widely known that improving the quality of healthcare is one of the most direct ways to address the significant health disparities between Aboriginal and Torres Strait Islander peoples and Australians of other descent. In 2008, the Council of Australian Governments (COAG) committed to ‘work together to achieve equality in health status and life expectancy between Aboriginal and Torres Strait Islander peoples and non-Indigenous Australians by the year 2023′ [1] (Hereafter, we use “Aboriginal” as a collective term, acknowledging the diversity of language and culture of Aboriginal and Torres Strait Islander peoples, as the First People and custodians of Australia). A core component of COAG’s ‘Closing the Gap’ strategy were measurable targets to monitor improvements in the health and wellbeing of the Aboriginal population. In response, there has been a rapidly expanding quest for information, reflected in a proliferation of quality improvement programs and introduction of key performance indicator (KPI) reporting [2]. In the Northern Territory, for example, gaps in quality of Aboriginal primary health care were identified following the introduction of Aboriginal Health Key Performance Indicators and performance reporting systems. These findings informed the Northern Territory’s continuous quality improvement strategy, which has been credited not only for its sustained use, but for the value of its data in strengthening health systems and improving quality of health care for Aboriginal peoples [3].

Reports on health care quality and health system performance, however, repeatedly lack information about Aboriginal health promotion programs. For example, health promotion—described as ‘activities designed to improve or protect health within social, physical, economic and political contexts’- is one of 68 performance measures included in the Australian Government’s Aboriginal and Torres Strait Islander Health Performance Framework [4]. Reporting on this measure is based on the number of health promotion interventions provided by clinicians and other health professionals. Information that could be used to monitor quality of Aboriginal health promotion is lacking. Cited reasons for this include suitability of indicators to measure and monitor quality, and limitations in data availability and quality [4].

In addition to improving reporting of the contribution that Aboriginal health promotion makes to ‘closing the gap’, there are calls for Aboriginal people and communities to become active partners in their health care delivery [5, 6]. COAG’s most recent Closing the Gap report makes stronger assertions to increase meaningful partnerships between all levels of governments and communities, in recognition that work to date is insufficient for meeting 2023 targets [7]. Studies evaluating Aboriginal people’s participation in health promotion have consistently concluded that community involvement enhances delivery and uptake of health programs [8–11]. However, the value of health promotion has yet to be fully realized because there remains insufficient evidence to confidently determine the impact on Aboriginal health and wellbeing [10, 11]. It has been suggested that by improving documentation of community participation strategies and processes, more successful strategies could be identified and replicated, thus strengthen the evidence base [10, 11].

Health information systems (HIS) have the potential to capture and share data that could improve quality and reporting of Aboriginal health promotion, including details of community participation. Firstly, by facilitating collection, documentation and organization of a vast array of information about health promotion in a structured and systematic way. Secondly, as a source of data to be analyzed and communicated in real-time for quality improvement and performance indicator reporting purposes. A HIS commonly used in hospitals and medical services is the Electronic Medical Record (EMR). EMRs, digital versions of the patient chart, contain information about patient medical and treatment history collected by and for clinicians, usually within a single healthcare institution. EMRs are valuable sources of data that providers can use in making decisions about health care delivery. Indeed, health services have sought innovative ways to utilize these data to report on and improve the quality health care and health system performance for Aboriginal Australians [12, 13]. EMRs and many other health information systems, however, are rarely designed or developed to capture, store or retrieve data about population-level services and activities. Currently, there is some evidence to suggest that health promotion and prevention could similarly benefit from health information systems [14, 15]. Yet, to our knowledge, there is no research on the potential use of these systems in the context of Aboriginal health promotion. Research into such technologies is challenging because HIS for recording and monitoring health promotion efforts are often created for individual organization’s internal purposes, without any public record of how it was designed, used or lessons learned [16].

Within this broader context, we report a study of Australia’s first investigation of a HIS designed for recording and storing information about Aboriginal health promotion. The Quality Improvement Program Planning System (QIPPS) was an innovative and unique online, project planning and evaluation system for health promotion and community development projects. From 2008 until 2019, when QIPPS was decommissioned and no longer available on the market, it was the centralized online system for recording information about health promotion programs delivered by Northern Territory Health (NT Health). We were interested in the feasibility of utilizing this information to report on the quality of Aboriginal health promotion. Specifically, our aims were to extract data stored in QIPPS to describe: (1) the scope of Aboriginal health promotion programs; (2) the quality of Aboriginal health promotion program planning, delivery and evaluation; and (3) community participation strategies and processes used in Aboriginal health promotion, using chronic disease prevention activities as exemplars programs. Thereby, we identify the benefits and limitations of HIS’ for health promotion and potential for secondary uses of stored data for quality improvement purposes.

## Study context

The Northern Territory (NT) is arguably Australia’s most challenging health service delivery environment. The NT has the highest proportion of Aboriginal Australian residents compared to other states in Australia. Approximately 30% of the total NT population identify as being Aboriginal peoples compared to 3% of the total Australian population [17], making NT Health the single largest provider of health services to Aboriginal peoples in Australia. About 90% of the NT Aboriginal population live in discrete, remote communities, where the delivery of health care is logistically challenging, hence more expensive, than in urban settings [18]. The gap in life expectancy between Aboriginal peoples and Australians of other descent is greater in the NT (14.4yrs for both males and females compared to 10.6 years for males and 9.5 years for females, nationally), and is increasing over time [1]. The cost of the Aboriginal health gap in the NT has been estimated at $16.7 billion [20].

The NT Aboriginal population experience a disproportionate burden of chronic disease linked to inactivity, diet, socio-economic disadvantage and access to primary health care services [19]. NT Health—the public healthcare system responsible for delivering clinical, primary health care and public health services to all Territorians – recognizes the critical role of health promotion and prevention in addressing these inequities and improving Aboriginal health outcomes. Health promotion is an ongoing strategic priority of NT Health [18, 22] and a core function in models of comprehensive primary health care [21]. However, in reality, a range of challenges influence health promotion delivery and its success in the NT, including the burden of acute care in Aboriginal communities, high workforce turnover, low stability and acute-oriented, temporary staffing [19, 23] together with the availability of information about, and capacity to report on, health promotion quality and effectiveness [10, 23, 24].

To overcome some of these challenges, NT Health has introduced over the past 10 years a range of initiatives. These have included: (i) a Health Promotion Strategic Framework [25]; (ii) introduction of the Quality Improvement Program Planning System (QIPPS); and (iii) participation in continuous quality improvement initiatives [26], including in health promotion specifically [27]. These initiatives have proved useful in guiding planning and implementation of health promotion programs across the NT’s diverse context, and there is some evidence of impact on health promotion quality [10, 24]. Previous assessments, however, have mainly been conducted at a community-level. There remains limited knowledge on the extent to which these initiatives are meeting Territory-wide strategic health promotion goals.

## Methods

### QIPPS: health information system for health promotion

Up until 2019, QIPPS was commercially available; hosted, maintained and supported by Infoxchange; a not-for-profit social enterprise with a focus on smart and creative use of technology to improve the lives of vulnerable people, driving social inclusion and creating stronger communities (see https://www.infoxchange.org/au).

Drawing from ‘best-practice’ community development principles, QIPPS was designed to guide practitioners, predominately public health, health promotion and community development workers, through designing, implementing, monitoring and evaluating diverse health promotion programs. QIPPS was not designed for recording data about individual patient information nor was it linked to health information systems, such as EMRs. QIPPS featured a series of tabs, a common feature for modern websites and databases, offering a navigation system for users. Tabs were arranged according to the main stages of the health promotion planning cycle (planning, implementation, and evaluation). Each tab included structured prompts to guide recording of information (mostly free text) related to each stage of the cycle. For example, a tab for “Needs Assessment” included prompts such as ‘describe the issue or problem the project aims to address’, ‘the population group most affected’, and ‘evidence to substantiate the rationale for proceeding with the project’. The “Evaluation” tab was divided into sections for recording findings of process, impact and outcome evaluations. Embedded in each tab were a wide range of supportive information including definitions, research material, references, website links, best practice models and frameworks.

QIPPS was also promoted as a mechanism for knowledge exchange. Users could export their program information as a Microsoft Word document to share with other project partners, and were able to search a growing body of community-based initiatives (QIPPS library).

In contrast to other health promotion systems which are typically created and used within an organization [16], QIPPS was Australia’s only fee-for-service commercially available, purpose-built HIS for health promotion. Organizations subscribed to QIPPS, with fees determined by number of total users.

Since 2008, NT Health subscribed to QIPPS with the intent of assisting public health staff (i) to design and deliver health promotion projects, and (ii) in documenting their health promotion efforts in a systematic and structured way. A 2017 internal survey identified between 320 and 380 public health staff had used QIPPS to record their health promotion work. Thus, QIPPS provided a potentially valuable, yet relatively unexplored, data source about health promotion in Aboriginal contexts.

### Study design

This study was a retrospective audit of health promotion projects recorded and stored in QIPPS. We included health promotion projects that: addressed chronic diseases, including mental health, environmental health, and/or risk factors (smoking, alcohol, nutrition, physical activity), designed to benefit Aboriginal people, families and communities, and that were recorded in QIPPS as delivered between 2013 and 2016. This selection of health promotion projects was made based on NT Health advice and knowledge of health promotion investment and information needs to inform future planning.

### Data collection and analysis

Our approach to data collection, analysis and reporting draws on a popular continuous quality improvement technique, known as audit and feedback. ‘Audit and feedback’ is a systematic process of gathering information about professional practice and then comparing this with explicit criteria (such as professional standards or targets) [28]. The gap between assessed performance and the criteria allows health services to target efforts on areas for improvement. ‘Audit and feedback’ is widely used by Aboriginal primary health care services to assess and improve health care quality [2, 29], including health promotion [9, 27].

**Fig. 1:**
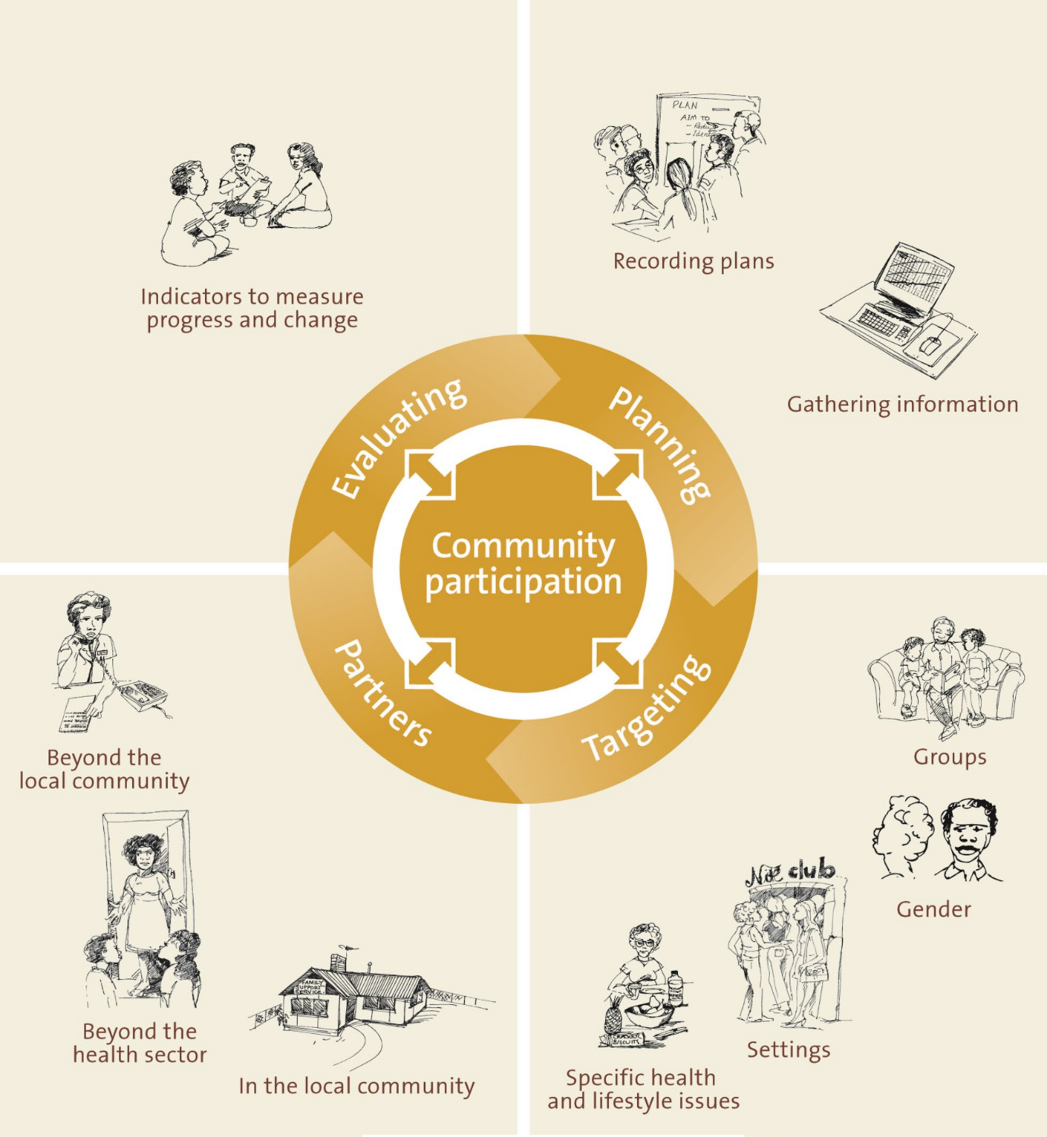
Indicators of Aboriginal health promotion quality

We used a previously validated, paper-based audit tool structured around five indicators of best practice Aboriginal health promotion. Indicators were identified by blending available best practice guidelines and practice-based evidence in Aboriginal health promotion [27]. The five headline indicators are (Fig. 1): Planning, Targeting, Community Participation, Partnerships, and Evaluation. Each headline indicator has several sub-indicators giving further insight into Aboriginal health promotion quality.

Data on scope and quality of Aboriginal health promotion were collected from information stored in the QIPPS online system. Project records were reviewed and checked for documentation of items specified in the audit tool and described using dichotomous (yes/no) and categorical variables. Categorical variables were not mutually exclusive. For example, ‘do records indicate whether community people participated in planning, implementation or evaluating this activity?’ If yes, select in what areas: ‘identifying the problem’, ‘determining or deciding strategies to address the problem’, ‘implementing the strategies’, ‘evaluating the activity’. This process was repeated for each project. To improve quality and consistency of data collection, an auditing protocol was used to guide data collection. The health promotion audit tool and protocol are available at https://www.menzies.edu.au/page/Resources/Health_Promotion_CQI_Tools/).

Four authors independently reviewed projects and recorded their findings in a purpose-built Microsoft Excel spreadsheet. The spreadsheet included each audit tool item. Simple frequency counts were calculated for dichotomous and categorical variables. In addition, we extracted text that described strategies and processes of community participation in health promotion projects. To ensure completeness of data and accuracy, three authors independently conducted audits on an initial sample of five projects. The lead author’s results acted as the ‘gold standard’ against which team members results were compared. Variations in results were discussed to determine reason for difference and strategies for enhancing data collection consistency. This included minor changes to the wording in the audit protocol and amendments to the data entry spreadsheet. Monthly meetings among co-authors were used to discuss and monitor emerging results. One co-author, not involved in the audit process, reviewed findings and interpretation.

## Results

### Scope of aboriginal health promotion projects in the northern territory

A total of 39 chronic disease prevention projects were included in the analysis. Most projects addressed nutrition (27 projects), followed by physical activity (7 projects) and mental health (including social and emotional wellbeing) (4 projects). Almost half (19/39) of the projects were considered once off (i.e. delivered only once and not expected to be done again). Five projects were continuous (i.e. delivered on a regular basis through the year e.g. monthly or weekly); and five were described as intermittent (e.g. delivered once a year, each year). Delivery frequency was unclear for ten projects. The type of health promotion strategies was dominated by health education (23 projects), followed by community action (19 projects), health information (18 projects) and strategies for creating supportive environments (14 projects).

### Quality of Aboriginal health promotion

Table 1 presents a summary of the aggregated data to report on indicators of Aboriginal health promotion quality. Most projects (34/39) included descriptions of planning processes; this included a clear statement of the project aim or goal (33/34) and details on the implementation strategies (31/34). Three quarters of projects (26/34) had documentation of the indicators or criteria to evaluate the project. Only 3 projects included a budget. Five projects had no documentation of planning processes.

**Table 1:** Summary of audit findings against indicators of Aboriginal health promotion quality

Audit tool items related to five best practice criteria	Number of projects with documentation of audit item* (total number of projects = 39)
1. Planning	**34**
that included:	
Aim	33
Strategies	31
People responsible for tasks	7
Timeframes	11
Indicators or evaluation measures	26
Budget	3
2. Targeting	**35**
That included:	
*Target group*	***35***
General population	16
Children (infants, preschool, primary school)	17
Adolescents and young adults	8
Adults	14
Elderly	5
Parents and families	11
*Gender*	***33***
Males	0
Females	4
Both males and females	26
*Setting*	***33***
Health centre	3
Community	28
Both health centre and community	2
*Health issue/topic*	***37***
Smoking	2
Nutrition or diet	27
Alcohol	2
Physical activity or exercise	7
Mental health/social and emotional wellbeing	4
3. Community participation	**20**
That included:	
Identifying needs	16
Determining strategies	11
Implementing strategies	7
Evaluating	4
4. Partnerships	**26**
That included:	
Outside agencies and organisations	26
Organisations beyond the health sector	15
5. Evaluation	**15**
That included	
Number of participants	12
Participant satisfaction	4
Changes in knowledge and understanding	6
Changes in skills and behaviours	3
Changes in policy and/or environments	3

Bold values indicate the number of projects with documentation of headline indicators

^*^Some projects included documentation of more than one audit item, therefore the total number exceeds the number of projects included in the study

In 35 of the 39 projects, there was a clear record of the ‘target group’, or, those who would benefit from the project. Most projects were designed for the “general community”, followed by “children”. There was a record of involvement of other organizations or agencies in 26 of the 39 projects, of which, 15 were with agencies or organizations beyond the health sector. Details about evaluation results were reported in approximately one third of projects (15/39); majority of the evaluation documentation was limited to a recording of participant numbers (12/15). The level of detail describing other evaluation findings were mixed, such as reporting changes in participant satisfaction (4/15), knowledge and understanding (6/15), skills and behaviours (3/15) or broader impacts on policy/environments (3/15).

### Recording of community participation in QIPPS projects

Documentation of community participation in health promotion planning, implementation and evaluation was reported in 20 of the 39 projects (see Table 1). Of the three phases, most of the recorded information pertained to the project planning phases (identifying need (16/20) and determining strategies (11/20)). Documentary evidence of community involvement during project implementation (7/20) or evaluation (4/20) was limited. In almost half of the projects, information describing community participation was not available or in insufficient detail, despite the QIPPS prompt to record “*How will the target group and community stakeholders be encouraged to actively participate and engage with the project?*”.

Table 2 includes examples of the unstructured text derived from information recorded by practitioners (QIPPS users); illustrating how strategies and processes of community participation are described in real world practice. The main strategy by which community participation happened was via consultation processes. Common consultation methods included community meetings, focus groups, surveys and interviews. Some project records included more detailed descriptions of how community participated than in others.

**Table 2:** Exemplar descriptions and number of projects that included a record of community participation strategies in QIPPS (n = 20 projects)

Identifying/determining need (16/20 projects)	Determining strategies (11/20 projects)	Implementing strategies (7/20 projects)	Evaluating strategies (4/20 projects)
After conducting a community consultation with 13 community women, they all expressed a desire to participate in group education around exercise and healthy eating	Clinic staff including Community Based Workers—can assist with community engagement and evaluation processes, undertake training in the healthy food sale	One community elder and a night patrol worker really liked the resource and they wanted to take Primary Health Network to introduce her to a different groups of women in the community to show them the resources and do education with them using the stories specially concerned for women who are pregnant and smoke	Adapt resource package based on community feedback as required
Community members in [community] expressed an interest in nutrition education and healthy cooking activities	A similar group was conducted in community which the women stated they all enjoyed, during the community consultation process	Activities and discussion were done in language	All "top end" public health nutritionists to offer and conduct takeaway monitoring
Particular community leaders have indicated that soft drink is something that they would like to help the community find a strategy to reduce consumption	Adapted workshop content and logistics based on CBW feedback in planning stages and previous TAFBALK evaluations	Strong Women Workers are employed by Health Development who are active in health promotion activities and will be crucial throughout planning and implementation	Prominent community figures will be involved in the evaluation and the re-development of The program
Clear communication from Primary Health Network to community stakeholders via official letter of invitation	Include SWSBSC CBWs in planning TAFBALK workshop content to share community knowledge needs, comment on existing knowledge base of CBWs and ensure workshop remains culturally relevant and appropriate	The shelf labels were placed at the store shelves. This was done by a group of 8 school children	Include opportunity in evaluation for CBWs to suggest ways forward and ideas for ongoing support following the TAFBALK workshop
Collaborate with the Takeaway Store in identifying the need	A new interview strategy, where key community members and traditional owners would be targeted. This was done by the local project staff member developing a list of individuals to approach	Participation in the day by assisting with preparation, setting up and engaging with the community by encouraging locals to try the SBT and pick children up	
Consult with community leaders about proposed project.-consultation with community leaders using semi-structured questions -conduct meeting/focus groups	sample label designs were discussed with community,	Healthy Tucker/Long life shelf labels on healthy foods at the store, went to store with students and supported them	

CBW = community-based workers; SWSBSC = Strong Women, Strong Babies, Strong Culture; TAFBALK = Talking About Feeding Babies and Little Kids

## Discussion

This retrospective audit of health promotion projects demonstrates the potential of a purpose-built, health information system to capture and share data that could be used to report, and improve, the quality of Aboriginal health promotion. At NT Health, QIPPS was used to record and store information about diverse health promotion programs. Encouragingly, details about planning aspects of health promotion, like community consultations, were recorded such that the information could be used for secondary analysis. However, documentation of information for implementation and evaluation phases was missing or described insufficiently. QIPPS provided a valuable source of information about Aboriginal health promotion in the NT, but data availability and quality were limiting factors for reporting on health promotion quality. To realize the systems’ full potential more needs to be done to support and encourage accurate records of practice – not only health promotion plans or intentions—but also what practitioners actually do. In particular, more details and improved documentation about ways of engaging community would allow for successful strategies to be identified and replicated in future work. This would enhance program success, strengthen the evidence base and contribution of health promotion to closing the gap in Aboriginal health inequities.

Australian reports on health care quality and system performance do not always capture and track health promotion performance measures. Moreover, current indicators of Aboriginal health care quality do not monitor and report on Aboriginal participation in health care delivery [3]. Few research studies of Aboriginal health promotion have assessed quality against indicators of best practice; for those that have done this, it is generally reported at a community-, and not health system level [10, 24]. Using a structured data extraction tool designed for health promotion quality improvement purposes, this study provided insight into the scope of health promotion and identified gaps in quality which could be used to target system level changes and improve Aboriginal health promotion efforts. The inclusion of a range of indicators to assess community participation in health promotion enabled a nuanced exploration of the different ways community are engaged as active partners throughout each phase of the health promotion cycle. For example, we identified that the most common strategy of community participation occurred during the planning phase, using consultation processes. Yet, information about strategies and processes of community engagement during implementation and evaluation phases were not documented or not of sufficient detail. Our previous research demonstrated that using indicators of Aboriginal health promotion quality within a continuous quality improvement framework enhances health system capacity for recording health promotion, and subsequently the availability and quality of data [10]. With further support for uptake and implementation of quality improvement in health promotion, demonstrable and sustained improvements in Aboriginal health promotion are feasible [30, 31].

As for information systems more generally [12, 13, 32, 33], a significant constraint in realizing the potential of QIPPS was data quality. Information about some elements of health promotion, particularly related to project planning, were more readily available, and reliably collected, such as stating project goals and objectives, identifying the target group and health issues to be addressed. Meanwhile, information about aspects of project implementation and evaluation, such as strategies for community participation, evaluation methods and reporting findings, were missing or inconsistently reported and therefore, less reliably collected for secondary analysis.

From a quality improvement standpoint, data standardization is critical for monitoring indicators and tracking performance over time. Records about health promotion practice predominately constitute prose-like narratives, or free text, invariably resulting in inconsistency in documented information. However, important insights about the quality of Aboriginal health promotion, such as community participation processes and strategies, will likely remain invisible if information is recorded in pre-specified formats or by applying strict documentation practices. Herein lies one of many challenges in designing information systems to support recording, collection, analysis and reporting of health promotion data for quality improvement and performance reporting, alongside health professionals’ planning and evaluation needs [14–16].

The extent of generalizability of our study findings should take into account: (i) data were based on recorded health promotion practice, which may underestimate breadth and depth of actual health promotion efforts; (ii) given the long-standing use of QIPPS in NT Health, the quality of data reported is likely to be better than for other Aboriginal health services and state/territory government health departments more generally in Australia. Furthermore, NT Health provided support and training for QIPPS users –Aboriginal and non-Aboriginal dedicated health promotion practitioners and non-dedicated health promotion staff including nutritionists and public health staff in hearing, oral and environmental health—thus, staff have a better understanding of the information system which is likely to influence quality of information entered in QIPPS; and (iii) several biases can arise auditing records of health service delivery, including experience and skills of the auditor/s; and the type of data extracted, influencing the reproducibility of quality indicator/s. A strength of the study was the iterative and team-based approach of Aboriginal and non-Aboriginal researchers, policy staff and health promotion practitioners working together.

Since completing this study, QIPPS was decommissioned by Infoxchange and is no longer available on the market. NT Health is currently transitioning from using QIPPS to an internal record management system. Study findings will inform the development of customized templates for documenting planning and evaluation of health promotion, and tailored workforce development initiatives to improve quality and accuracy of data entry and recording.

We hope this study will also inform future discussions and design of health promotion information systems and promote the potential for secondary uses of stored data for performance reporting and quality improvement purposes. To reap the full potential of health promotion information systems, we recommend continuous use of quality improvement approaches, such as tracking quality indicators using an audit and feedback technique. A continuous quality improvement approach could encourage, monitor, and reward accurate reporting of indicators. This would enhance health information system functionality, the capability of practitioners to use these systems effectively for planning and evaluation, and for monitoring improvements in quality of health promotion.

## Conclusion

This first Australian study of a purpose-built, health promotion information system demonstrates the potential for utilizing stored data to report on quality of Aboriginal health promotion. More should be done to encourage accurate recording of information about health promotion practice, particularly findings of evaluations and how community are engaged throughout the health promotion project cycle. This would allow the more successful strategies to be identified and replicated to enhance health promotion success, and ultimately improve the health and life expectancy of Aboriginal peoples. Testing and improving the validity and reliability of indicators of Aboriginal health promotion quality is an important area for future research. More specific attention to the development and use of information systems in health promotion should contribute to a more comprehensive understanding of the quality of health services and programs for Aboriginal Australians.

## Data Availability

The dataset analyzed during the current study are not publicly available because the information system has been decommissioned and the product is no longer available on the market. Data are however available from the corresponding author upon reasonable request and with permission of NT Health.

## Abbreviations

CBW: Community-based workers
EHR: Electronic health record
NT: Northern Territory
QIPPS: Quality Improvement Program Planning System
SWSBSC: Strong women, strong babies, strong culture
TAFBALK: Talking about feeding babies and little kids
